# Supplementary material for: Relationship between outpatients’ sociodemographic and belief characteristics and their healthcare-seeking behavioral decision-making: Evidence from Jiaxing city, China
Source: PLoS One. 2022 Jun 30;17(6):e0270340. doi: 10.1371/journal.pone.0270340 (PMC9246228; doi:10.1371/journal.pone.0270340)
Supplement: S1 File — (DOCX) [file pone.0270340.s001.docx]

1. Detailed theoretical basis of characteristics’ selection

During the design and research process, we undertook a detailed literature review, but could not include it in our main text due to limits on the length of the article. In the supplemental file, we added the following theoretical basis regarding the main selection of characteristics.

In 1968, Andersen first proposed the behavioral model of medical service utilization, which represented a systematic research into patients’ healthcare-seeking behavioral decision-making. The structural framework of the model comprises the following elements. 1) Environmental factors: They primarily include the external environment, such as natural, political, economic, and medical system. 2) Population features: They predict and explain the use of medical services through three variable levels, affecting health outcomes and service satisfaction. ➀Predisposing factors: They can be categorized into personality factors, social structure, and health beliefs. Personality factors, such as age, gender, and other demographic factors, indicate people’s need for medical services based on their physiological characteristics. Social structure is measured by a series of indicators that reflect the individuals’ social status and their ability to deal with problems. Common indicators include education, profession, race, social network, social relations, and culture. Health beliefs refer to people’s attitudes, values, and knowledge of health and medical services. They affect people’s perception of the use and demand intensity of medical services. ➁Enabling factors: They promote or hinder people’s ability to use medical services, including personal, family, and community resources. Personal resources include income, medical insurance, and time required to reach medical institutions among others. Family resources include family structure and function. Community resources are mainly supplier factors. ➂Need (Personal perceived medical service needs): It is the direct cause of medical service utilization. Affected by social factors and health beliefs, it reflects how people view their health and functional status, disease symptoms and pain, and worry about their health. When necessary, self-assessment (subjective needs) and assessment (objective needs) can be carried out. 3) Health behavior: It includes personal medical and health service utilization behavior. 4) Health outcome: It can be evaluated using three aspects: self-perception of health status, health status assessed by professional medical personnel, and patient satisfaction [1].

Using the above framework, many scholars have examined the influencing factors of individual aspects, such as Andersen pointed out that symptoms themselves are also a form of social construction. Patients’ perceptions and interpretations of symptoms are affected by their social and cultural backgrounds [2]. In addition, some researchers believe that a relatively “disadvantaged group” is more inclined to self-diagnosis, choosing a hospital closer to their residence, or a private hospital [3]. Some scholars in China have indicated that patients tend to choose a high-level medical institution given sudden or severe illness; greater financial resources enable patients to actively seek health care services, and the improvement in cultural literacy facilitates patients paying more attention to their own health [4].

Moreover, Behavioral Decision Theory posits that the emergence of any decision is inseparable from three factors: the contextual factors underlying the decision, the features of the individual’s beliefs, and the individual’s preference structure [5,6]. According to the theoretical background of Behavioral Decision Theory, the American psychologist Daniel Kahneman and the Israeli behavioral finance scientist Amos Tversky [7] proposed a new theory of behavioral decisions, Prospect Theory, by introducing psychology to economics. Prospect Theory is based on the individual’s actual state of decision-making; it focuses on the psychological reasons for their behavior. According to Prospect Theory, subjective systematic bias and individual risk preference directly affect the decision-making process and the patient’s interpretation and value judgment of the expected goal, thus affecting their healthcare-seeking behavioral decision-making. Subjective systematic bias varies with cognitive ability and disease severity. For example, when patients face uncertain prospects at decision-making nodes, the more serious they consider their illness, the greater their fear, and the greater the extent their decision-making will be affected by their risk preference, resulting in less informed or objectively based healthcare-seeking behaviors [7]. In contrast, if the expected result is highly certain, patients will comprehensively consider factors including medical technology, economy, and convenience of medical treatment, and their healthcare-seeking behaviors will tend to be more reasonable [8].

According to the theoretical framework described above and the data available from the questionnaires, we examined six patient sociodemographic characteristics：gender, age, profession, birthplace, location of residence, and having medical insurance. Additionally, using the Prospect Theory paradigm and health beliefs or outcomes, we evaluated patients’ self-recognition of the severity of their disease (serious, medium, or minor). It is important to clarify that patients’ financial status is very important in our research. However, to respect patients’ privacy, their profession was used as a proxy (e.g., farmer, industrial worker, staff or civil servant, freelancer or individual owner) given that, in China, profession and income or social stratum are correlated to some extent [9].

Reference:

1. (America) Wallinsky.(1999). Sociology of Health (Sun, M.H., Trans). Beijing China, BJ: Social Sciences Literature Press.
2. Andersen, R.S., Vedsted, P., & Olesen, F. (2009). Patient delay in cancer studies: a discussion of methods and measures. BWC Health Service Research,9(189).

[3] Puck, D.C., Beukers, Ron, G.M., & Kemp, M. V. (2014). Patient hospital choice for hip replacement: empirical evidence from the Netherlands. Eur J Health Econ, 15, 927-936.

[4] Guo, W.Q., Wu, Y.A., & Yao, Z.Y. (2010).Analysis of medical behaviors and influencing factors of rural chronic disease patients. Chinese Primary Health Care, 24 (1), 65-67.

[5] George, L., & Jennifer, L. (2003).The Role of Affect in Decision Making: Handbook of Affective Science (pp.256-298). Oxford England, Oxford: Oxford University Press.

[6] Herbert, G. (2007).A framework for the unification of the behavioral sciences. Behavioral and Brain Sciences, 30(1), 1-61.

[7] Daniel, K., & Amos, T. (1979). Prospect Theory: an analysis of decision under risk. Econometrica, 47(2), 263-292.

[8] Huang, H. (2010). Study on health seeking behavior of community residents (pp.14-15).[Unpublished Master's thesis]. Jiangsu University, China.

[9] Xueyi Lu. (2018). Research Report on social structure of contemporary China. Beijing China, BJ: social sciences academic press.

1. Details of population participation rate of medical insurance in Jiaxing

Zhu Chen, Minister of Health of China, announced at the second China Health Forum in 2011 that the medical insurance coverage rate of Chinese residents had increased from about 15% in 2000 to nearly 95% at the end of 2010, covering 1.27 billion people [1]. However, this is the whole country’s data, which cannot be used to assume that all regions are the same. In addition, in 2010 and after more than 10 years, most parts of the country have still not ensured that an outpatient’s settlement of medical insurance in a different city be affiliated to their origin city. In other words, for example, although the permanent nonnative outpatients who saw a doctor in Jiaxing had medical insurance in their native city, they could not be reimbursed by Jiaxing’s system of medical insurance. The following Table 1 presents changes in the number and insured rate of permanent residents in Jiaxing, including the number of Jiaxing’s native and nonnative residents who have participated in medical insurance in recent years.

Table 1. The number and insured rate of permanent residents in Jiaxing

|  | Permanent native population in Jiaxing | Permanent nonnative population in Jiaxing | Total permanent population in Jiaxing | Total Jiaxing-  insured population  in Jiaxing | Jiaxing-  insured population of Jiaxing’s native | Total  Jiaxing-  insured rate | Jiaxing-  insured rate of  Jiaxing’s  native | Jiaxing-  insured rate of  Jiaxing’s  nonnative |
| --- | --- | --- | --- | --- | --- | --- | --- | --- |
| 2016 | 352.1 | 109.3 | 461.4 | 248.7 | 208.5 | 53.9% | 59.2% | 36.8% |
| 2017 | 356.4 | 109.2 | 465.6 | 261.8 | 216.5 | 56.2% | 60.8% | 41.5% |
| 2018 | 360.4 | 112.2 | 472.6 | 403.1 | 354.7 | 85.3% | 98.4% | 43.2% |
| 2019 | 363.7 | 116.3 | 480.0 | 413.3 | 362.1 | 86.1% | 99.6% | 44.1% |
| 2020 | 367.6 | 172.5 | 540.1 | 423.5 | 366.6 | 78.4% | 99.7% | 33.0% |

Data sources: the statistical yearbook on the official website of Jiaxing Statistics Bureau, the annual medical insurance express on the official website of Jiaxing Medical Insurance Bureau, and internal data of the government.

Table 1 shows that for the total permanent population in Jiaxing (including native and nonnative), the rate of participating in Jiaxing local medical insurance has increased yearly from 53.9% in 2016 to 78.4% in 2020; however, the rate of nonnative residents’ participating in Jiaxing local medical insurance was low every year, no more than 50%, with some of them participating in the medical insurance in their cities of origin. According to the staff of Jiaxing Medical Insurance Bureau, Jiaxing did not implement the reimbursement of outpatient’s medical insurance to be affiliated to different provinces and cities until December 2020, implying that although the nonnative population in Jiaxing had medical insurance in their city of origin, it was generally impossible for outpatients in Jiaxing to realize its use before 2020. This study’s questionnaire survey was conducted in July 2018, and the outpatient data extracted from Jiaxing population health platform mainly came from 2017–2019. In 2017, the insured rate of the total permanent population of Jiaxing was only 56.2%, and that of Jiaxing’s native residents was only 60.8%. In addition, in the field survey, it was found that even if several outpatients have medical insurance, in view of the high starting line of medical insurance reimbursement, or the high percentage of the billed charges being paid by the patients themselves, some outpatients, especially those who do not spend too much money to see a doctor occasionally, simply pay at their own expense, or stop participating in the insurance the next year. According to the “statistical express on the development of medical security in 2020” recently released by the National Medical Insurance Bureau, the number of urban and rural residents participating in basic medical insurance in 2020 decreased by 8.06 million, or 0.8% lower compared with 2019 [2], similarly, the participation rate of the total permanent population of Jiaxing also decreased from 86.1% in 2019 to 78.4% in 2020. In addition, according to Ping Liu [3], while the proportion of medical insurance reimbursement in China has increased significantly, the participation rate is declining. According to the statistics of the relevant departments of Zhejiang Province, at the end of 2016, the cooperative medical fund for urban and rural residents in Nanhu District of Jiaxing City reported a cumulative loss of 7.59 million yuan. This could seriously affect the development of urban and rural medical reform, resulting in the phenomenon of “cooking out” of medical security in the next few years.

Reference:

1. Zhu Chen.(2011). China's basic medical insurance coverage rate is nearly 95%, covering 1.27 billion people. Decision making reference of hospital leaders, 17(03).
2. National Medical Insurance Bureau.(2021). Statistical bulletin on the development of medical security in 2020.
3. Ping Liu. (2018). Problems and Countermeasures of medical insurance for urban and rural residents. Rural economy and technology, 29 (08): 216.
4. The basis of defining minor or general diseases in population health platform

First, comprehensive clinical diagnosis includes not only the diagnosis of etiology, pathoanatomy, and pathophysiology, but also the classification and staging of diseases, as well as the diagnosis of complications and associated diseases. The classification or staging of “severe” or “middle and late stage” was not reflected in the diagnostic names of “minor diseases” or “common diseases” selected from Jiaxing population health platform, and there were no complications and associated diseases.

In addition, in the study of Jiaxing population health platform, the author subjectively defined “minor diseases” and “common diseases” based on her experience as an attending physician, and the definitions were also corroborated by the policy documents issued by the State or the Medical Insurance Bureau. This judgment was based on a statement by Shanchang Xu, deputy director of the Medical Reform Office of the State Council, who pointed out that the “serious disease” mentioned in the “Guidance on carrying out serious illness insurance for urban and rural residents” [1] was not a medical disease concept. The document simply did not distinguish serious diseases according to the types of diseases, instead, it determined them according to the comparison between the high medical expenses caused by serious diseases and the economic affordability of urban and rural residents. As long as the medical expenses paid by the insured exceed the local annual per capita income, they can enjoy serious illness insurance [2]. At present, there is no clear and unified definition of “serious illness” of the “serious illness medical insurance” program in China. According to the disease definition of major disease insurance jointly formulated by China Insurance Industry Association and China Medical Association [3], 25 serious diseases have been identified (the specific figures are still being updated), including malignant tumors (excluding some early malignant tumors), acute myocardial infarction, sequelae of stroke (permanent dysfunction), and major organ transplantation or hematopoietic stem cell transplantation (allogeneic transplantation) among others.

Thus, the main text (on page 15) emphasizes that the definition of “minor diseases” or “common diseases” has been excluded according to the scope specified by the State in the serious disease insurance program. Further, it emphasizes that this study focused on the medical decision-making behavior of outpatients, and their social consciousness level was the basis for determining “minor disease” or “common disease,” rather than scientific clinical diagnosis.

Reference:

1. Xinhua News Agency.(2012, Aug 30th). The guiding opinions on carrying out serious illness insurance for urban and rural residents. Retrieved Aug 30th, 2012, from http://www.gov.cn/jrzg/2012-08/30/content_2213783.htm
2. China.com.(2012, Sep 4th). Medical reform office interprets "serious illness medical insurance", which does not distinguish between diseases.Retrieved Sep 4th, 2012, from <http://roll.sohu.com/20120904/n352321089.shtml>

[3] China Insurance Industry Association and China Medical Association.(2020). Specification for use of disease definition of major disease insurance (revised in 2020).

1. Selection of medical institutions for questionnaire survey

At present, the three-level referral system has not been strictly implemented in the outpatient clinics in China, that is, people can choose any level of hospital (level one, two, or three) for their first outpatients’ service.

When selecting medical institutions for the questionnaire survey, our principle was that the proportion of outpatients’ visiting the hospital throughout the year should be as large as possible, and the proportion of the selected hospitals should try to conform to the distribution of real outpatients in Jiaxing throughout the year. If a hospital was selected at random, the sample data would not have represented the real overall data.

Among the many medical units in Jiaxing, we have selected six, including five tertiary and secondary hospitals. These five hospitals constitute the main force of outpatient services in the city, accounting for 79.7% of the total number of outpatient services in the city in 2017; the annual outpatient visits of all primary hospitals and other secondary hospitals accounted for 15.2% and 5.1%, respectively. Because other secondary hospitals account for very little (5.1%), we did not choose the corresponding institution, however, we chose Nanhu Community Health Center, which is one of the representative primary hospitals.

1. Implementation details of decision tree analysis

Artificial intelligence is a branch of computer science. It attempts to understand the essence of intelligence and produce a new machine that can respond similar to human intelligence. Research in this field includes robots, language recognition, image recognition, natural language processing, and expert system. Machine learning is a subset of artificial intelligence. It obtains the information required human beings through algorithm learning from data. Machine learning algorithms can be categorized as supervised, unsupervised, semi-supervised, and reinforcement learning. Decision tree used in our research is a non-parametric, supervised machine learning method [1]. And decision tree can summarize decision rules from a series of data with features and labels, and its basic process conforms to the strategy of divide-and-conquer; thus, it is ideal for explanation and visualization [2]. Moreover, it can deal with nonlinear characteristics, and the interaction between variables is considered. Compared with regression analysis, the decision tree has more explanatory power, and if the classification boundary of sample points is nonlinear and can be simulated by dividing the feature space into rectangles, the decision tree is better than logistic regression [2].

Regarding the minimum number of samples required for a decision tree analysis, there is no consensus yet, however, the number of training set samples required for machine learning is positively related to the quality and number of features in the data set. A common rule of thumb is that a model usually needs 10 times more data than its degree of freedom, where the latter is generally regarded as the number of features in the dataset [3]. Moreover, in machine learning, a learning curve is a collection of data points (xj, yj) that describes how the accuracy of a classifier (yj) is related to training sample sizes (xj), where j = 1 to m, with m being the total number of instances. There is evidence that yj would reach 85% or higher accuracy when using a relatively small sample size of 200 for the learning curve; after 200, the accuracy rate increases slowly [4]. We determined a minimum sample size of 195 (close to 200) for this study.

Regarding the specific algorithm of the decision tree model, we chose the C4.5 classifier. Lemnaru found that in the classifiers of machine learning, MLP and C4.5 classifiers are less affected by the imbalance, while the SVM classifier generally performs poorly in imbalanced problems [5]. In the modeling procedure by PyCharm (V 2019.3.1), we found the information gain rates of C4.5 for gender and self-report of the disease’s severity were c. 0.1% and smaller; the contributions were significantly small due to cross-entropy loss. Additionally, from a common-sense perspective, self-report of the disease’s severity (this time to see a doctor) is unrelated to the questionnaire items (i.e., the patient’s general behaviors). Therefore, considering that the model has other features and that more samples are needed, we conducted another decision tree model without these two features and reanalyzed it using the C4.5 classifier.

Moreover, we applied the holdout method to solve the overfitting problem, which was using estimation and validation steps (stratified sampling to divide training and validation sets, at a ratio of c. 1:8 from the 195 samples). The estimation step grew the initial tree with the training set, and the validation step developed the optimal tree pruning rule with the validation set. The pruning rule used was Cost Complexity Pruning (post-pruning). Post-pruning is based on the whole decision tree built before the prune, it investigates the non leaf node from bottom to top. If replacing the subtree corresponding to the node with leaf node can improve the generalization performance, replace the subtree with leaf node. Investigating each non leaf node is regarded as a once tree prune, finally, the final model is selected according to the smallest error rate on the validation set [6]. After the 13th prune, the error proportion in the validation set was 20.5%; accordingly, we chose this as the final decision tree model. Finally, the model was drawn using Matplotlib of PyCharm (V2019.3.1) .

Reference:

1. Zhou ZH. Machine Learning. Beijing China: Tsinghua University Press; 2016.China.
2. Announcing CSDN Blogs. [cited 17 Sep 2018]. In: CSDN Blogs [Internet]. Deep understanding of machine learning. Available from: https://blog.csdn.net/hy592070616/article/details/81628956.
3. Announcing CSDN Blogs. [cited 12 Dec 2020]. In: CSDN Blogs [Internet]. Fundamentals of machine learning. Available from: https://blog.csdn.net/fengdu78/article/details/111087503.
4. Rosa L. Figueroa Qing Zeng-Treitler, Sasikiran Kandula, Long H Ngo. Predicting sample size required for classification performance . BioMed Central. 2012; 12(1).
5. García S, Herrera F. Evolutionary undersampling for classification with imbalanced datasets: Proposals and taxonomy. Evolutionary computation. 2009; 17(3): 275-306.
6. Announcing Jianshu Blogs. [cited 11 May 2019]. In: Jianshu Blogs [Internet]. CCP Pruning. Available from: <https://www.jianshu.com/p/0e4a34a41d36>.
