# Supplementary material for: Relationship between outpatients’ sociodemographic and belief characteristics and their healthcare-seeking behavioral decision-making: Evidence from Jiaxing city, China
Source: PLoS One. 2022 Jun 30;17(6):e0270340. doi: 10.1371/journal.pone.0270340 (PMC9246228; doi:10.1371/journal.pone.0270340)
Supplement: S1 Appendix — (DOCX) [file pone.0270340.s002.docx]

**Appendices**

**1. The Questionnaire**

**The Questionnaire of Health-Seeking Behavioral Decision-making**

Gender_______; Age______; Profession_______; Birthplace________; Residency Location_________; Having or not having medical insurance_____（Yes or No）; The degree of self-recognition of the disease ________(serious, medium or minor).

1) Your general habit of seeing a doctor (single choice) —————————( )

A. Minor and serious diseases to go to large hospitals.

C. Minor diseases go to small hospitals, and serious diseases go to large hospitals.

2) Will you choose to see a doctor in Shanghai or Hangzhou? (single choice) —( )

A.Yes, the medical level is higher than Jiaxing

B. No, the medical level in Jiaxing is enough

C. No, Jiaxing’s hospitals have medical experts for consultation from Shanghai and Hangzhou

D. No, it's too troublesome or expensive to go to a larger hospital in Shanghai and Hangzhou

**2. Tables**

| **Variable** | n | % | Mean | Std. Deviation | **Variable** | n | % | Mean | Std. Deviation |
| --- | --- | --- | --- | --- | --- | --- | --- | --- | --- |
| **Gender** |  |  | 0.56 | 0.04 | **Medical insurance** |  |  | 0.21 | 0.03 |
| male (0) | 85 | 43.59 |  |  | yes (0) | 155 | 79.49 |  |  |
| female (1) | 110 | 56.41 |  |  | No (1) | 40 | 20.51 |  |  |
| **Age** |  |  | 1.07 | 0.06 | **Degree of self-recognition of the disease** |  |  | 0.71 | 0.05 |
| <40 (0) | 54 | 27.69 |  |  | minor (0) | 76 | 38.97 |  |  |
| 40-60 (1) | 73 | 37.44 |  |  | medium (1) | 100 | 51.28 |  |  |
| >60 (2) | 68 | 34.87 |  |  | serious (2) | 19 | 9.75 |  |  |
| **Profession** |  |  | 1.81 | 0.07 | **Residency location** |  |  | 0.27 | 0.03 |
| famer (0) | 18 | 9.23 |  |  | Jiaxing (0) | 143 | 73.33 |  |  |
| work (1) | 57 | 29.23 |  |  | near Jiaxing (1) | 52 | 26.67 |  |  |
| staff or civil servant (2) | 64 | 32.82 |  |  |  |  |  |  |  |
| Freelancer or individual owner (3) | 56 | 28.72 |  |  |  |  |  |  |  |
| **Birthplace** |  |  | 0.87 | 0.06 |  |  |  |  |  |
| Jiaxing (0) | 82 | 42.05 |  |  |  |  |  |  |  |
| near Jiaxing (1) | 57 | 29.23 |  |  |  |  |  |  |  |
| from a distance (2) | 56 | 28.72 |  |  |  |  |  |  |  |

1) **Table3. The data about seven characteristics of the outpatients in the questionnaire (N=195)**
