# Supplementary material for: Relationship between outpatients’ sociodemographic and belief characteristics and their healthcare-seeking behavioral decision-making: Evidence from Jiaxing city, China
Source: PLoS One. 2022 Jun 30;17(6):e0270340. doi: 10.1371/journal.pone.0270340 (PMC9246228; doi:10.1371/journal.pone.0270340)
Supplement: Attachment — Submitted filename: Manuscript Rev 2-editor_comments_suggestions.docx [file pone.0270340.s005.docx]

**The relationship between outpatients’ sociodemographic and belief characteristics and their healthcare-seeking behavioral decision-making: evidence from Jiaxing city, China**

Mingming Yu ^1*,#a^, Zan Yang ^2,#a^, Lemin Shi^3,#b^

^1^ Department of Economics and Management, Tongji Zhejiang College, Jiaxing, Zhejiang, China

^2^ Department of Science, Tongji Zhejiang College, Jiaxing, Zhejiang, China

^3^ Health Education Center, Jiaxing Municipal Health Commission, Jiaxing, Zhejiang, China

^#a^ No 168, Business Road, Jiaxing City, Zhejiang Province, China

^#b^ No.486, Wenqiao Road, Jiaxing City, Zhejiang Province, China

* Mingming Yu

Email: [yigemeng_1@163.com](mailto:yigemeng_1@163.com) (MM)

**Abstract**

**Background**

China established the Tiered-network Healthcare Delivery System (THDS) in 2005 to address the disproportionate number of patients attending tertiary hospitals relative to primary- or secondary-care institutions. Although the reported number of outpatients visiting tertiary hospitals has been slowly decreasing, numerous patients still choose to visit them regardless of the severity of their disease. To effectively implement the THDS in the future, this article explored the relationship between patients’ sociodemographic and belief characteristics and their healthcare-seeking behavioral decision-making in China.

**Methods**

Data obtained through questionnaires were analyzed using decision tree and logistic regression models to explore outpatients’ characteristics and medical decision-making using comprehensive feature data. In addition, further statistical analyses were conducted on the outpatient data obtained from the regional population health platform in Jiaxing, China.

**Results**

The decision tree model revealed that whether outpatients have medical insurance is the primary factor guiding their healthcare-seeking behaviors, with those without medical insurance being more likely to choose small hospitals to treat minor diseases. For those with medical insurance, profession becomes the main factor, with industrial workers being more inclined to choose small hospitals for minor diseases. The logistic regression analyses revealed that outpatients without insurance and who were not freelancers or individual owners were more likely to choose small hospitals for minor diseases. Further statistical analysis of the data from the Jiaxing population health platform showed that, for minor or general diseases, outpatients without medical insurance and employed as farmers tended to choose secondary and primary hospitals over tertiary hospitals.

**Conclusion**

The three analyses yielded consistent results: in China, having/not having medical insurance and patients’ profession are the most important factors guiding outpatients’ healthcare-seeking behaviors. Accordingly, we propose that the government should focus on economic reforms to increase outpatients’ visits to small hospitals as well as diagnosis-related groups payment of medical insurance to decrease the admittance of patients with minor diseases in large hospitals. Meanwhile, the government should correct patients’ belief prejudice of selecting hospitals through corresponding publicity.

**Keywords:** behavioral decision-making; outpatients’ characteristics; Tiered-network Healthcare Delivery System.

**Introduction**

China’s current medical system is the product of its socialist (mixed socialist and managed capitalist would be better?)  economic system [1]. To date, public and private hospitals have coexisted, with the former being the majority (by the end of 2019, public hospitals’ beds accounted for 72.5% of the total number of hospital beds [2]). As per the national medical service system, hospitals are classified as primary, secondary or tertiary based on their size and the level of health care provided [1]. For instance, tertiary hospitals provide the highest level of treatment and specialization. These institutions have not only received consistent policy attention and support through financial subsidies [3], talented personnel, and loans, but they have also made full use of the market economy system reforms during the past 30 years. Moreover, they (all tertiary?) have retained considerable profits (what does ‘profit’ mean for public hospitals?) by adding a 15% mark-up on drug sales and setting high prices on high-tech diagnostic tests [4-5].

Although this situation has been gradually changing since China’s 2009 Health Policy Reform [4-5], which includes a zero-mark-up drug policy and reduced prices for diagnostic tests, tertiary hospitals have created a monopoly advantage. (I think more explanation is needed regarding how and why tertiary hospitals gained a monopoly advantage? The 2009 reform only applied to them, giving them a cost advantage?) Thus, many patients choose to be treated in large tertiary hospitals, regardless of whether they have serious or minor diseases [3]. In fact, by the end of 2019, 23.6% of Chinese outpatients and 39.4% of inpatients were concentrated in tertiary hospitals, which only account for 0.27% of all hospitals, despite the average outpatient and inpatient cost ratio in tertiary, secondary, and primary hospitals being 3:2:1 (337.6:214.5:142.6, Unit: RMB) and 4:2:1 (13.7:6.2:3.3, Unit: Thousand RMB)[2], respectively.

Consequently, large (tertirary?) hospitals are often overcrowded and Chinese patients increasingly experience difficulties in and high costs of obtaining medical services. To address this problem, in 2005, China’s administration established the Tiered-network Healthcare Delivery System (THDS) [6], (what did which proved difficult to implement because of the liberalization and marketization of patients choices of medical treatment and the small price gaps between primary, secondary, and tertiary hospitals [7-8]. (To me, this section makes it sound like a 2005 THDS addressed a problem created by the 2009 zero mark-up policy. I think the previous two paragraphs need to be clarified) In 2013, a survey of six cities in China revealed that 62.2% of patients felt uncomfortable choosing primary care institutions for their first visit to a doctor [9], however, the number of patients visiting these institutions is slowly increasing [10]. Since the THDS was not being implemented satisfactorily, China has strengthened the THDS’ reform since 2017 [11]. This included price modifications, which led to significant reductions (until 2019, it has fallen to 23.1% after 2017) in the number of outpatient visits to tertiary hospitals in Beijing city [12].

The reduced number of outpatients visiting tertiary care institutions following the 2017 THDS reform suggests the following question: What type of patients would choose to visit primary and secondary hospitals instead of tertiary institutions? Answering this question is tantamount to drawing a “portrait” of the patient by considering comprehensive features, which, if undertaken correctly, could improve public medical administrators’ understanding of patients’ medical decision-making. This, in turn, could facilitate the efficient equitable distribution of medical resources and effective guidance of patients consistent with standards of care.

The abovementioned portrait would reflect the relationship between the patients’ sociodemographic and belief characteristics and their healthcare-seeking behavioral decision-making. While this relationship has been analyzed in previous studies regarding specific diseases or populations [13,14,15], it has not been studied in the context of effective implementation of the THDS. Moreover, although there is evidence from existing research that uses multivariable logistic regression and other statistical tests to analyze the factors that affect patients’ behavioral intentions [16,17,18], there is minimal information on using technology (e.g., machine learning classifier) to examine the relationship between the studied variables. To fill these research gaps, we conducted a comprehensive empirical analysis of healthcare-seeking behavioral decision-making based on a hybrid theoretical framework.

**Materials and Methods**

**Theoretical model**

The hybrid theoretical framework used in this research was built by combining behavioral the behavioral Anderson and prospect decision making theories. The hybrid decision making model was estimated using machine learning techniques (machine learning is an estimation method, the text seems to say an estimation technique has some substantive theoretical content, which is a strong and surprising claim that needs to be explained and justified. I think theoretical framework and estimation method used to analyze the data need to be carefully distinguished unless you have a strong argument to the contrary) Andersen’s behavioral model of health services use behavioral decision theory, prospect theory, and several important factors influencing healthcare-seeking behavioral decision-making that have been generally recognized by scholars in recent years, such as economics, distance from hospitals, and self-recognition of the disease.[19,20,21]. Andersen’s behavioral model involves systematic research, including population features, such as 1) predisposing factors: They can be categorized into personality factors (age, gender, and other demographic factors), social structure (education, profession, race, and social network among others), and health beliefs. (people’s attitudes, values, and knowledge of health and medical services). These predisposing factors affect people’s perception of the use and demand intensity of medical services. 2) enabling factors. They promote or hinder people’s ability to use medical services, including personal, family, and community resources. Personal resources include income, medical insurance, and time to arrive at medical institutions among others. 3) need: (I don’t’ see a description or definition of ‘need’, the word is following by a description of factors that affect it, please supply a definition for description) It is affected by social factors and health beliefs, and reflects how people view their health and functional status, experience disease symptoms and pain, and worry about their health[22].

Prospect theory is based on the individual’s actual state of decision-making. According to prospect theory, subjective systematic bias and individual risk preference directly affect the decision-making process and the patient’s interpretation and value judgment of the expected goal, thus affecting their healthcare-seeking behavioral decision-making. Subjective systematic bias varies with cognitive ability and disease severity [23].

Conversely, machine learning (machine learning is an estimation technique, the language seems to imply it is statement about the content or structure of the Anderson and prospect theories) theories are concerned with features and labels (I think readers not familiar with machine learning will have trouble understanding the terms ‘features’ and ‘labels’ I think you need two sentences explaining what they mean in the context of this study). In this study, the main machine learning task was to extract useful features and construct the mapping from features to labels; in other words, to draw a portrait.

According to the hybrid theoretical framework (I am confused, if Anderson’s theory includes prospect theory, then prospect theory is not per se an additional theory used in addition to Anderson’s theory, so not sure why you call it hybrid. Until there is an explanation how machine learning has substantive theoretical content, is just a way to calculate estimates, so it can’t be part of a hybrid theory either. I think a lot more explanation is needed here to justify your apparent lumping the theory and estimation method together as a ‘hybrid theoretical framework’, I think Anderson’s model itself can be characterized as a ‘hybrid theoretical framework’ Is that what you mean?), we selected seven patient characteristics that comprehensively reflect sociodemographic and belief features based on the research paradigms of behavioral theories. The labels in the questionnaire were health-seeking behavioral decisions about the THDS. (I think you should add more detail on how the labels are related to the health seeking deciisons -those are choice of tertiary versus other hospitals? And put this sentence right after you first mention labels’)

(Shouldn’t the section below be included under Survey and Questionnaire Design? If you think not, then provide language in the Survey and Questionnaire Design section more clearly linking the description of the patient demographics in this section to the description of the survey)

**Measures Describing Patient characteristics**

We examined seven (self-reported?) patient characteristics: gender (male or female), age (<40, children or youth; 40–59, middle age; >59, old age), profession, birthplace (in Jiaxing, near Jiaxing, or distant from Jiaxing), location of residence (in Jiaxing, near Jiaxing), having medical insurance (yes or no), and patients’ self-recognition (would ‘self-report’ be better, I think ‘self-recognition’ is not a standard term, so its meaning needs to be explained and use justified) of the severity of their disease (serious, medium, or minor). “In Jiaxing” or “near Jiaxing”, Jiaxing includes the two districts, two counties, and three county-level cities. Some patients were not only working, but also living in the central urban area, that is, the urban districts, and were characterized as living “in Jiaxing” in our questionnaire survey, while those characterized as living “near Jiaxing” generally worked and lived in the surrounding counties and county-level cities. “Distant from Jiaxing” denotes cities other than Jiaxing. Outpatients living “near Jiaxing” generally use buses and cars to reach the hospitals in the central urban area, and experience longer transportation time (usually 30 minutes to 1 hour by car, and longer by bus). (Therefore, compared with outpatients living in the central urban area, who face shorter transportation time, (usually less than 30 minutes by car), those living in the surrounding counties and cities are inconveniently far away. (Were the terms ‘in Jiaxing’, ‘near Jiaxing’ and ‘distant from Jiaxing’ categories that the survey subjects could choose from, or assigned by the authors?)

Profession is used as a proxy for income Financial status is very important in our research. However, to respect patients’ privacy, their profession was used as a proxy (e.g., farmer, industrial worker, staff or civil servant, freelancer or individual owner), given that, in China, profession and income are correlated to some extent [24].

**Survey and Questionnaire Design**

Therefore, we administered our questionnaire to outpatients in Jiaxing’s several hospitals, and Jiaxing is a representative, medium-sized city in China. The Equilibrium and Demand Theory (this is not a standard economic term used in the US or Europe that I know of, I think you need to use another term or explain it, so you just mean ‘Demand for health care’?) attributes the need for regional medical services, which is clearly manifested in outpatients’ healthcare-seeking behaviors, to demographic and economic factors [25]. Surveying outpatient departments instead of specific communities and units better reflects the randomness and reality of a city. Therefore, we administered our questionnaire to outpatients presenting at the outpatient clinics Jiaxing’s several hospitals.Jiaxing is a representative, medium-sized city in China.

The questionnaire consisted of two parts. The first part collected data about the seven representing sociodemographic and belief characteristics of the outpatients (I think you should either move the section describing the demographic features to here, or refer to that section here if you don’t move it), while the second addressed patients’ healthcare-seeking behavioral decision-making regarding the THDS using the following question. The respondents were asked about their general behaviors when seeing a doctor; specifically, whether they attended a large hospital regardless of the severity of the disease (option A), or whether they visited a large or small hospital for serious or minor disease, respectively (option C).

The sample size was 200 patients, calculated using the following formula:$N=Z^{2}\times\left(P\times\left(1-P\right)\right)/E^{2}=196$ (196 should be 1.96?), where, Z score is 1.96 when the degree of confidence is 95%, and *P* is the probability value, generally 0.5, and E is the sampling error rate taken as 7%. (Reference for the sample size formula that you used?) In addition, the questionnaire was administered in the outpatient departments of five (public and private, or only one of them?) tertiary and secondary hospitals and one primary hospital in Jiaxing. The tertiary and secondary hospitals included Jiaxing First Peoples Hospital, Jiaxing Traditional Chinese Medicine Hospital, Jiaxing Second Peoples Hospital, Jiaxing Armed Police Hospital, and Jiaxing Third Peoples Hospital. Their outpatient visits in 2017 separately accounted for 24.7%, 23.2%, 21.2%, 5.6%, and 5% of the total number of outpatient visits in Jiaxing, respectively, for a total of 79.7% of all outpatient visits. In contrast, visits to primary hospitals in Jiaxing accounted for 15.2% of the total number of outpatient visits in the city, while visits to other secondary and township hospitals accounted for 5.1% [26]. Based on the proportion of outpatient visits in the city, we distributed the 200 respondents as follows: 168 respondents across the five tertiary and secondary hospitals and 32 to Nanhu Community Health Center (a primary hospital, we surveyed this hospital to represent all primary hospitals in Jiaxing), with the ratio of respondents being 5.3:1 (roughly in line with the ratio of 79.7%:15.2% = 5.2:1). Given the small proportion of “other” hospitals (i.e., other secondary or township hospitals), we did not include them in our research.

To ensure that the sample was randomly selected under the same condition, in given hospitals, all questionnaires were administered simultaneously (July 5, 2018) and the respondents were selected using a (simple?) random sample based on outpatient ID number (the sample frame was the hospital’s administrative patient admission system, right? I think you need to describe the sample frame from which the random sample was drawn), without excluding any disease. The outpatients were instructed to complete the questionnaire based on their general health-seeking behaviors. Only three and two outpatients in the tertiary and primary hospitals, respectively, refused to participate. Thus, 195 valid (you mean complete? What does ‘valid’ mean in this context) questionnaires were obtained. The survey process was approved and supervised by the Municipal Health Commission of Jiaxing, and all participants signed written informed consent forms before completing the questionnaire. I think the highlighted section below should be in the Results section) The baseline details of the 195 respondents are presented in Table 1.

**Table 1. Seven outpatients characteristics (N=195)**

| **Variable** | n | % | **Variable** | n | % | **Variable** | n | % |
| --- | --- | --- | --- | --- | --- | --- | --- | --- |
| **Gender** |  |  | **Medical insurance** |  |  | **Residency location** |  |  |
| male (0) | 85 | 43.59 | yes (0) | 155 | 79.49 | in Jiaxing  (0) | 143 | 73.33 |
| female (1) | 110 | 56.41 | no (1) | 40 | 20.51 | near Jiaxing (1) | 52 | 26.67 |
| **Birthplace** |  |  | **Degree of self-recognition of the disease** |  |  | **Age** |  |  |
| in Jiaxing  (0) | 82 | 42.05 | minor (0) | 76 | 38.97 | <40 (0) | 54 | 27.69 |
| near Jiaxing (1) | 57 | 29.23 | medium (1) | 100 | 51.28 | 40–60 (1) | 73 | 37.44 |
| from a distance(2) | 56 | 28.72 | serious (2) | 19 | 9.75 | >60 (2) | 68 | 34.87 |
| **Profession** |  |  |  |  |  |  |  |  |
| famer (0) | 18 | 9.23 |  |  |  |  |  |  |
| worker (1) | 57 | 29.23 |  |  |  |  |  |  |
| staff or civil  servant (2) | 64 | 32.82 |  |  |  |  |  |  |
| freelancer or  individual  owner(3) | 56 | 28.72 |  |  |  |  |  |  |

**Decision tree analysis**

The outpatients’ responses regarding their general healthcare-seeking behaviors were classified into groups using a decision tree: a non-parametric, supervised machine learning method. (I would include a reference here to an introduction to decision tree machine learning methods) One of this technique’s potential disadvantages is over-fitting [27]; thus, we used the machine learning test ( ‘machine learning test’ is not a standard term to my knowledge and can mean several very different things. I think you mean that you used a cross-validation procedure to develop optimal pruning rules, a test data set to measure out-of-sample predictive performance of the estimated model, and then chose the model with the best predictive performance, and it looks like you did that in this paper. Readers unfamiliar with machine learning will think that you did a formal hypothesis test, but no result of a formal hypothesis test appears in the results.). set to prune the initial decision tree, thereby reducing the risk of over-fitting and improving the model’s generalization ability. (I don’t think readers who understand machine learning at all will like the passage highlighted in yellow above. I recommend giving more detail, and putting in a supplemental file, or just giving a reference. Solving the overfitting problem using estimation and validation steps. The estimation step grows the initial tree, the validation step develops optimal tree pruning rules usually using cross-validation. then the tree is regrown using the whole data set using the pruning rules developed in the validation step. The test data set is NOT used to estimate the tree or develop pruning rules. When the sample size is large enough to do the estimation and validation steps on a subset of the data, a test data set is set aside to measure the predictive performance of the tree using data that was NOT used for either estimation or validation. I think the text needs to be clarified, or give a very brief description and provide an introductory reference)

I think that the text highlighted in blue below should be put into a supplemental text for deails on the estimation of the decision tree. I don’t think many readers will understand the use of MPL, SVM and C4.5 for building decision trees. I suggest adding the following reference for readers unfamiliar with these terms:

Moreno-Ibarra, Villuendas-Rey, Lytra et al. Classification of Diseases Using Machine Learning Algorithms: A Comparative Study. A Comparative Study. Mathematics 2021, 9, 1817. https://doi.org/10.3390/math9151817

Regarding the minimum number of samples required for a decision tree analysis, there is no consensus yet, however, the number of training set samples required for machine learning is positively related to the quality of and number of features in the data set. A common rule of thumb is that a model usually needs 10 times more data than its degree of freedom, where the degree of freedom is generally regarded as the number of features in the data set [28]. In addition, in machine learning, a learning curve is a collection of data points (x_j_, y_j_) that describes how the accuracy of a classifier (y_j_) is related to training sample sizes (x_j_), where j = 1 to m, with m being the total number of instances. There is evidence that y_j_ would reach 85% or higher accuracy when using a relatively small sample size of 200 for the learning curve; after 200, the rate of accuracy increases slowly [29]. We determined a minimum sample size of 195 (close to 200) for the present study.

For machine learning classifiers, the imbalance in the number of category variables will affect the accuracy of classification. Data may be categorized depending on their imbalance ratio (IR), which is defined as the relation between the majority class and minority class instances, using the following formula:$IR=\frac{N^{-}}{N^{+}}$, where, $N^{-}$ and $N^{+}$ is the number of instances belonging to the majority and minority class, respectively. According to Lemnaru, an IR above 9 represents a high IR in a data set, due to the fact that ignoring the minority class instances by a classifier, supposes an error of 0.1 in accuracy, which has poor relevance[30]. Also, MLP and C4.5 (in our research, we used C4.5) are less affected by the imbalance, while SVM generally performs poorly in imbalanced problems[31]. Sample descriptive statistics are reported in Table 1. Among the 195 valid samples, the seven characteristics IR in the sample data are not above 9.

The open-source software PyCharm (need a citation for the software program here) was used to analyze the questionnaire data from the 195 samples using the C4.5 algorithm [27] (This reference uses seme version of C4.5, but I can’t find the term in the text, so I doubt it will be a useful reference for the reader, I suggest you use the reference I supplied above) and to obtain information gain rates of the seven outpatients’ characteristics. The information gain rates for gender and self-recognition of the severity of the disease were c. 0.1% and smaller; the contributions were very small due to cross-entropy loss. Additionally, from a common-sense perspective, self-recognition of the severity of the disease (this time to see doctor) is unrelated to the question in the questionnaire (i.e., the patient’s general behaviors). Therefore, considering that the model has other features and that more samples are needed, we conducted another decision tree model without these two features and reanalyzed it using the C4.5 algorithm.

The initial prototype of the decision tree was obtained by selecting features from the largest to the smallest cross-entropy loss and growing the branch-leaf nodes from the top to the bottom. Because of the over-fitting shortcoming, we used the machine learning test data set (how large wat the test data set?) for pruning, which was randomly selected by PyCharm at a ratio of c. 1:8 from the 195 samples; the method of pruning was Cost Complexity Pruning (post-pruning) [32]. Before pruning, the decision tree’s proportion of errors for the test data set was 23%. After the thirteenth prune, the error proportion was 20.5%; accordingly, we chose this as the final decision tree model. Finally, the model was drawn using Matplotlib of PyCharm (Fig 1).

**Fig 1. The final decision tree model**

**Logistic regression analysis**

A decision tree model can be verified using statistical tests, which makes it possible to explain and confirm the reliability of the model [33]. Therefore, we used logistic regression analysis to verify the results of our decision tree model. This allowed us to compare similarities and differences, or advantages and disadvantages of the two methods. (I think there is a conflation of sensitivity/robustness analysis and validation in the text highlighted in yellow above. A validation suggests that you used logistic regression to generate predictions in a test data set and then compared the two models’ predictive performance, but I don’t see that you did that in the results. So, I think the logistic regression is better characterized as a sensitivity/robustness analysis, in that the two model specifications were consistent.)

The dependent variable comprised participants’ responses to the question of whether they attended a large hospital regardless of the severity of their disease (option A, which was coded as 0) or whether they attended a large hospital for serious diseases and a small hospital for minor diseases (option C, which was coded as 1). The independent variables were the outpatients’ sociodemographic and belief characteristics, which were dummy-coded. The model was specified as follows: *logit*(*p*_i_) = φ + αN_i_ + βO_i_ + γP_i_ + δQ_i_ + εR_i_ + ιS_i_ + θT_i,_ where, N is gender, O is age, P is profession, Q is birthplace, R is residency location, S is having or not having medical insurance, and T is the self-recognition of the severity of the disease. The remaining letters and i represents coefficients and the order of outpatients, respectively. Backward-stepwise regression analysis was used with the variables with *p* < 0.05. The data were analyzed using Stata V.13. (Need a citation here)

**Analysis of the data from the population health platform**

( I think you need to explain in more detail how, in what way, this analysis validates the decision tree analysis here, at the beginning of this section. I think how this analysis work is fairly clear in the Results section, but I think you need to explain it with similar clarity here. Also I think a sentence or two that describes how these data differ from the data used in the decision tree analysis is needed) In order to verify the reliability and validity of the data collected using questionnaires, we analyzed additional data available through the Jiaxing’s population health platform of outpatients who had visited the city’s hospitals. We randomly selected two sample datasets consisting of 12,464 outpatients (dataset 1) that visited tertiary and 10,237 outpatients (dataset 2) who visited the city’s secondary or primary hospitals during 2017–2019, respectively. These patients were all suffering from ailments such as cold, hypertension, diabetes, and bronchitis. Neither were the classification and staging of severe or middle and late stage reflected in the diagnostic names, nor were there any complications and associated diseases. We considered these diseases "minor or general", because, first, they were excluded from the scope of the serious disease insurance scheme specified by the State [34], and second we focused on outpatients’ social belief level, rather than scientific clinical regulation.

We compared the proportion of outpatients with and without medical insurance as well as the proportion of farmers and non-farmers across the two datasets (we could only obtain information about profession in terms of farmer or non-farmer) using Pearson’s chi-square test, and found the differences to be statistically significant (*p* < 0.05).. Moreover, we compared the results from the Jiaxing’s population health platform and the decision tree model regarding the relationship between outpatients’ characteristics and their compliance with the THDS. The data were analyzed using Stata V.13

**Results**

I think you should include Table 1 and discussion here

**Decision tree analysis**

(I think the language here sounds more like an interpretation (for example, using ‘illustrates’. I think you need a more objective and complete description of the estimated tree, with references to the specific nodes for readers unfamiliar with decision trees) Figure 1 illustrates three findings regarding outpatients’ healthcare-seeking behaviors. First, whether outpatients have medical insurance is a primary factor in their behavioral decision-making, with those without medical insurance being more likely to visit small hospitals for minor diseases and large hospitals for serious diseases. Second, among those who do have medical insurance, outpatients’ profession becomes the main factor influencing their decision-making. That is, staff or civil servants as well as freelancers or individual owners are more likely to visit large hospitals for both minor and serious diseases, while industrial workers are more inclined to visit small hospitals for minor diseases and large hospitals for serious ones. Third, if outpatients were farmers residing near Jiaxing, they were more likely to choose small hospitals for minor diseases and large hospitals for serious ones.

**Logistic regression analysis**

The last model was *logit*(*p*_i_) = 0.206 + 0.946S_i_ - 0.824P-3_i_. (P-3 was a dummy variable representing freelancer or individual owner). The likelihood ratio was $\chi^{2}=$ 9.92, *p* = 0.007, therefore, the overall model was statistically significant. Regarding S and P-3 independent variables, their *p* values were 0.017 and 0.016, respectively. In logistic regression, another important value is the odds ratio (OR); the OR values for S and P-3 were 2.576 and 0.439, respectively. This means that among individuals with the same profession, the OR of patients without medical insurance (who were more likely to choose option C than A in the question of questionnaire) was 2.576 times that of those with medical insurance. Further, within the same condition of having or not having medical insurance, the OR of patients who were not freelancers or individual owners (who were more likely to choose option C than A) was 1/0.439 = 2.28 times that of those who were freelancers or individual owners (Table 2).

**Table 2. Results of logistic regression** ^a^

|  | Odds Ratio | Std. Err. | z | P>\|z\| | [95% Conf. Interval] |
| --- | --- | --- | --- | --- | --- |
| Prof-3 | 0.438 | 0.149 | -2.42 | 0.016 | 0.225 0 .855 |
| insurance | 2.576 | 1.020 | 2.39 | 0.017 | 1.185 5.599 |
| constant | 1.228 | 0.221 | 1.14 | 0.253 | 0.863 1.748 |

^a^ Number of obs = 195, LR chi2 (2) = 9.92, Prob > chi2 = 0.0070

**Analysis of data from the population health platform**

After removing duplicate patients and invalid samples, 850 and 830 cases from dataset 1 (the tertiary hospitals) and dataset 2 (the primary and secondary hospitals), respectively, were separately selected. Comparing the two datasets of patients, it was found that there was no significant difference in gender and age (*p* > 0.05), however the incidence of having medical insurance ($\chi^{2}=10.802$, *p=*0.001) and non-farmer’ profession in dataset 1 ($\chi^{2}=52.062$, *p=*0.000) was higher than that in dataset 2. We then analyzed whether the farmer profession was related to having or not having medical insurance. In dataset 1, under the condition of no gender and age difference between the farmer and non-farmer group (*p* > 0.05), the incidence of not having medical insurance and being a farmer was higher than that of non-farmer patients ($\chi^{2}=27.221$, *p=*0.000; Table 3).

We concluded that for minor or general diseases, outpatients without medical insurance and employed as farmers were more likely to choose primary and secondary hospitals than tertiary hospitals, which was mostly consistent with the aforementioned results from the decision tree model. This difference may be because of the farmer or non-farmer profession, as the proportion of farmers with medical insurance is lower than that of other professions.

**Table 3. Comparison of outpatients’ target data between the group of tertiary hospitals and the group of primary and secondary hospitals groups**

| Target | In the tertiary hospital group  （*n*=850） | In the primary and secondary hospital group（n=830） | The value of $\chi^{2}$ | *P value* |
| --- | --- | --- | --- | --- |
| Male / female（number of cases） | 385/465 | 374/456 | $\chi^{2}$=0.009 | 0.923 |
| Children or youth (<40) /middle age (40-59) /old age (>59) （number of cases） | 423/180/247 | 449/171/210 | $\chi^{2}$=3.764 | 0.152 |
| With medical insurance / without medical insurance （number of cases） | 542/308 | 464/366 | $\chi^{2}$=10.802 | 0.001 |
| Farmer / non-farmer（number of cases） | 301/549 | 439/391 | $\chi^{2}$=52.062 | 0.000 |
| Target | Farmers in the tertiary hospital group  （*n*=301） | Non-farmers in the tertiary hospital group  （*n*=549） |  |  |
| Male / female（number of cases） | 123/178 | 262/287 | $\chi^{2}$=3.692 | 0.055 |
| Children or youth (<40) /middle age (40–59) /old age (>59) （number of cases） | 144/65/92 | 279/115/155 | $\chi^{2}$=0.749 | 0.688 |
| With medical insurance / without medical insurance （number of cases） | 160/141 | 390/159 | $\chi^{2}$=27.221 | 0.000 |

**Discussion**

With the goal of informing policy to effectively implement the THDS in China in the future, we explored outpatients’ healthcare-seeking behavioral decision-making and its relationship with their characteristics reflecting sociodemographic and belief. Accordingly, we collected data about outpatients’ characteristics and decision-making using questionnaires and analyzed them using a decision tree model and logistic regression. In addition, to verify our conclusions, we randomly selected real outpatient data from the regional population health platform for further statistical analysis.

( I think the text highlighted in yellow below should go into a supplemental file)

A decision tree can summarize decision rules from a series of data with features and labels, and its basic process conforms to the strategy of divide-and-conquer; thus, it is ideal for explanation and visualization. In addition, it can deal with nonlinear characteristics, and the interaction between variables is considered. Compared with regression analysis, the decision tree has even more explanatory power, and if the classification boundary of sample points is nonlinear and can be simulated by dividing the feature space into rectangles, the decision tree is better than logistic regression [35]. We used a decision tree’s machine learning method to draw “portraits” of outpatients based on their sociodemographic and belief characteristics, which provided detailed decision routes that the logistic regression could not supply. However, since the logistic regression analysis produced consistent results. In this study, the decision tree and logistic regression revealed similar results as the existing research[36], which were also found to be consistent with the actual data from the population health platform of Jiaxing City.

First, the results from the three methods revealed that having/not having medical insurance was the primary factor that guided the outpatients’ decision regarding which medical institution they visited: patients with medical insurance and minor disease were more likely to choose large hospitals, while patients without medical insurance and minor diseases were more likely to choose small hospitals. If the latter’s choices were for economic factors, those who had medical insurance and abandoned it were even more because of economic reasons. It is noteworthy that, in recent years, according to Ping Liu[37],the proportion of medical insurance reimbursement (not sure what this means, could you clarify? Do you mean a higher proportion of medical expenditure is reimbursed by insurance payments?) in China has increased significantly, however, the participation rate (in insurance programs?) has declined. This is also evident from the participation rate of the total permanent population of Jiaxing that has decreased from 86.1% in 2019 to 78.4% in 2020[38]. This shows that for residents who usually do not see a doctor or occasionally visit one for a minor illness, it is not cost-effective to pay the insurance premium compared with the proportion that can be reimbursed.

Moreover, the analysis revealed that, among patients with medical insurance, their profession, which is representative of social class in China, was the second most influential factor guiding outpatients’ healthcare-seeking behaviors. For example, staff and civil servants, as well as freelancers and individual owners have greater advantages than farmers and industrial workers in terms of economic power and individual capabilities. Therefore, such patients will choose large hospitals with better diagnosis and treatment expertise, regardless of whether they have minor or serious diseases. In contrast, farmers and industrial workers are more inclined to avoid seeking the highest standard of care.

A similar conclusion can also be drawn from the analysis of the data from the population health platform: outpatients who were employed as farmers rather than non-farmers were more likely to choose primary and secondary hospitals than tertiary hospitals. Professional differences was as same as the differences of having/not having medical insurance. In China’s cities, a considerable number of migrant workers are actually farmers in their place of origin. In recent years, although the medical insurance coverage rate of Chinese residents has been greatly improved compared with that of more than 10 years ago[39], and migrant workers may have medical insurance in their places of origin, they still do not receive medical insurance because the cross regional reimbursement of medical insurance has not yet been realized between different provinces and cities. The analysis of the population health platform revealed a gap in the availability of medical insurance between farmers and non-farmers in tertiary hospitals: better paying professions were more likely to be able to afford insurance. These findings are in line with international research showing that income is an important determinant of medical accessibility, especially in countries that lack universal medical insurance and predominately rely on private medical insurance [40].

Economic factors were found to be the most important for outpatients’ healthcare-seeking behavioral decision-making in China, despite having or not having medical insurance or belonging to a specific profession. As to patients’ beliefs, we can see from the data of the questionnaire: 19 outpatients whose self-recognition were serious diseases all chose large hospitals to see a doctor at the day we surveyed, of which 13 chose the best two tertiary hospitals in Jiaxing, and none chose community hospital. So, in China, regarding patients belief, there is significant difference in medical quality between large and small hospitals; thus, patients are willing to choose large hospitals for both minor and serious diseases. However, due to financial limitations, some patients who do not have medical insurance or who are industrial workers or farmers will choose to be transferred to small hospitals to treat their minor diseases. Based on these findings, we propose that the fundamental way to solve outpatients’ overcrowding large hospitals is to reduce current discrepancies among Chinese hospitals’ quality of diagnosis and treatment.

However, at least in recent years, it has been difficult to narrow the gap of medical quality between large and small hospitals. Since 2017, the THDS reforms in China have attempted to increase the coverage of elementary medical insurance population and change the ratio of medical insurance reimbursement: a reduced reimbursement ratio in large hospitals and an increased ratio in small hospitals [12]. According to our research results, these reforms would increase the number and diversity of patients in small hospitals, who are currently mostly farmers and industrial workers, which can strengthen the construction of small hospitals. However, the economic means would not decrease the number of patients in large hospitals, who are mostly wealthy people working as freelancers or individual owners.

This indicates that the effective implementation of the THDS policy is limited by patients’ freedom to choose which hospital or doctor to visit. In the future, the government needs to formulate reform plans for healthcare providers, such as payment methods for medical insurance. At present, medical insurance payment in China is mainly based on service items, while in developed countries it is based on disease types. There has also been a growing interest in using diagnosis-related groups (DRGs) payment to reimburse inpatient care worldwide [41]. In Zhejiang Province, China, for example, DRGs payment started in January 2020, with medical insurance being paid according to the administrative coordination area [42]. This can increase competitiveness among hospitals in the coordination area and guide patients with serious diseases to large hospitals, diverting patients with minor diseases to small hospitals.

In addition, according to the last decision tree model for farmers with medical insurance, diverting patients to medium or small hospitals is easier to achieve for outpatients living near Jiaxing. Long distances and inconvenience are factors outpatients consider when they seek medical treatments. This is consistent with the findings in the United States [43], Australia [44], and Turkey [45], which have confirmed a negative relationship between distance and readmission rate, and a positive relationship between distance and length of hospital stay. This also suggests that medical regional administrators could strengthen outcomes by building primary and secondary hospitals near urban cities to retain more patients and to reroute patients to these hospitals.

Our findings further revealed that the belief of outpatients was an important factor influencing their health-seeking decision. Primary and secondary hospitals have proven to be competent when dealing with minor or general diseases, however, people’s belief leads to prejudice, causing them to prefer visiting tertiary hospitals under better economic conditions. Therefore, government departments need to correct this bias of residents. They can guide them by disclosing the treatment performance of the second and primary hospitals, and publicize more the effective treatment of chronic and general diseases in these hospitals, rather than just publicizing the treatment results of tertiary hospitals for difficult and rare diseases.

Finally, through this application research, we found that due to the lack of data cleaning, standardized processing, and algorithm analysis suitable for the platform, it was difficult to carry out effective analysis at the big data (‘big data’ has not been defined, and is not a standard term, need to choose another word or explain what it means) level. For example, the platform research was carried out by random sampling, and the profession data were imperfect. So, access to good health system data remains one of the biggest barriers in developing effective policies in the area, next we should enhance the building of the population health platform.

**Acknowledgments**

∙We would like to thank Jiaxing Science and Technology Bureau to fund this research and thank Jiaxing Municipal Health Commission to assist the survey arrangement in hospitals we chose.

∙We would like to thank Editage (www.editage.com) for English language editing.

**References**

1. Gu X. The great transformation of medical policy in New China in the past 70 years: towards the complementary embedding of administration, market and community governance. China Health Insurance*.*2019;10 (38).
2. Statistical bulletin of health development in China in 2019. National Health Commission of China. 2020 Jan 20 [Cited 2020 June 8]. Available from:http://www.nhc.gov.cn
3. Sun L. Key points, difficulties and solutions of urban medical and health system reform in the context of healthy China. China Health Law. 2020; 28(01): 92-95.
4. Fu H, Li L, Yip W. Intended and unintended impacts of price changes for drugs and medical services: evidence from China. Soc Sci Med. 2018; 211:114–22.
5. Wang M, Yu GL, Huang YM. Study on the obstacles and Countermeasures of the implementation of the tiered-network healthcare delivery system. Health Economics Research. 2020; 2:57-63.
6. Yang N, Shen LY, Shu TH, Liao SJ, Peng Yi, Wang JH. An integrative method for analyzing spatial accessibility in the hierarchical diagnosis and treatment system in China. Social Science & Medicine. 2021; Forthcoming.
7. McCollum R, Chen L, Chen XT, et al. Experiences with primary healthcare in Fuzhou, urban China, in the context of health sector reform: a mixed methods study. Int J Health Plann Manage. 2014; 29:107–26.
8. Ding L, Wu J. The impact of China's national essential medicine policy and its implications for urban outpatients: a multivariate Difference-in-Differences study. Value Health. 2017; 20:412–419.
9. Tang C, Luo Z, Fang P, Zhang F. Do patients choose community health services (CHS) for first treatment in China? Results from a community health survey in urban areas. J Community Health. 2013; 5:864–72.
10. Song HY, Zuo X, Cui CS , Meng K. The willingness of patients to make the first visit to primary care institutions and its influencing factors in Beijing medical alliances: a comparative study of Beijing’s medical resource-rich and scarce regions. BioMed Central. 2019; 19(1).
11. State affairs office. Key tasks of deepening medical and health system reform in 2017. 2017 April 25 [cited 10 May 2017]. Available from: http://health.people.com.cn/n1/2017/0510/c14739-29264911.html
12. Zhu D , Shi X , Nicholas S , et al. Impact of China's healthcare price reforms on traditional Chinese medicine public hospitals in Beijing: an interrupted time-series study. BMJ Open. 2019; 9(8):e029646.
13. Ma, GX, Gao W, Lee S. Health seeking behavioral analysis associated with breast cancer screening among Asian American women. Int J Womens Health. 2012; 4:235-43.
14. Muhammad UF. [Mother education and child health outcomes: Evidence from Pakistan Demographic and Health Survey 2012-13](https://kns.cnki.net/kcms/detail/detail.aspx?filename=1019042955.nh&dbcode=CDFD&dbname=CDFD2019&v=). Doctoral dissertation, Shandong University, China. 2019. Available from: https://kns.cnki.net/kns8/defaultresult/index
15. Arnold EA, Fuller S, Kirby V, Steward WT. The impact of medicaid expansion on people living with HIV and seeking behavioral health services. Health Affair (Project Hope). 2018; 37(9).
16. Md Nazmul Hasan, M Sheikh Giash Uddin. Women empowerment through health seeking behavior in Bangladesh: Evidence from a national survey. South East Asia Journal of Public Health. 2016; 6(1).
17. Zhao MY, Liu HY, Qu SJ, He L,Campy Kathryn S. Factors associated with parental acceptance of influenza vaccination for their children: the evidence from four cities of China. Human Vaccines & Immunotherapeutics. 2021;17(2).
18. Zhou Q, Zhao Campy,Wang. Parental perceptions of human papillomavirus vaccination in central China: the moderating role of socioeconomic factors. Human Vaccines & Immunotherapeutics. 2019; 15:7-8.
19. Rosenberg M, Hanlon N. Access and utilization: a continuum of health service environments. Social Science and Medicine. 1996; 43(6): 975-983.
20. Andersen RS, Vedsted P, Olesen F. Patient delay in cancer studies: a discussion of methods and measures. BWC Health Service Research. 2009; 9(189).
21. Xing HY, Shen Y, Zhao HJ. Correspondence analysis of rural residents' health seeking behavior and its influencing factors. Chinese Rural Health Service Administration.2002; 22 (5):12-15.
22. (America) Wallinsky. Sociology of Health (Sun, M.H., Trans). Beijing China, BJ: Social Sciences Literature Press; 1999.
23. Daniel K, Amos T. Prospect Theory: an analysis of decision under risk. Econometrica. 1979; 47(2): 263-292.
24. Lu XY. Research Report on social structure of contemporary China. Beijing China, BJ: social sciences academic press; 2018.
25. Zhang LL, Hu SL, Wei Y. Analysis on the needs, demands and influencing factors of medical services for urban and rural residents in the region. China Health Economics. 2002; 3:36-39.
26. Jiaxing Municipal Bureau of Statistics. Jiaxing Statistical Yearbook-2017. Beijing China, BJ: Chinese Statistics Press; 2017.
27. Taeho Jo. Machine Learning Foundations: Supervised, Unsupervised, and Advanced Learning. Berlin German: Springer Press; 2021. (pp.141-165).
28. Announcing CSDN Blogs. [cited 12 Dec 2020]. In: CSDN Blogs [Internet]. Fundamentals of machine learning. Available from: https://blog.csdn.net/fengdu78/article/details/111087503.
29. Rosa L. Figueroa Qing Zeng-Treitler, Sasikiran Kandula, Long H Ngo. Predicting sample size required for classification performance . BioMed Central. 2012; 12(1).
30. Lemnaru C, Potolea R. Imbalanced Classification Problems: Systematic Study, Issues and Best Practices. In: Zhang R, Zhang J, Zhang Z, Filipe J, Cordeiro J. (eds) Enterprise Information Systems. ICEIS 2011. Lecture Notes in Business Information Processing. 2012; vol 102. Springer, Berlin, Heidelberg.
31. García S, Herrera F. Evolutionary undersampling for classification with imbalanced datasets: Proposals and taxonomy. Evolutionary computation. 2009; 17(3): 275-306.
32. Announcing Jianshu Blogs. [cited 11 May 2019]. In: Jianshu Blogs [Internet]. CCP Pruning. Available from: <https://www.jianshu.com/p/0e4a34a41d36>.
33. Announcing CSDN Blogs. [cited 12 Aug 2019]. In: CSDN Blogs [Internet]. On the advantages and disadvantages of decision tree. Available from: <https://blog.csdn.net/weixin_41540084/article/details/99325672>.
34. Health.sohu.com. Medical reform office interprets "serious illness medical insurance", which does not distinguish between diseases. China.org.cn. 2012 Sep 4 [cited 2013 May 8]. Available from: https://health.sohu.com/20130508/n375234178.shtml
35. Announcing CSDN Blogs. [cited 17 Sep 2018]. In: CSDN Blogs [Internet]. Deep understanding of machine learning. Available from: https://blog.csdn.net/hy592070616/article/details/81628956.
36. Lee Yoonju, Kim Heejin, Lee Yesul, Jeong Hyesun. Comparison of the Prediction Model of Adolescents' Suicide Attempt Using Logistic Regression and Decision Tree: Secondary Data Analysis of the 2019 Youth Health Risk Behavior Web-Based Survey. Journal of Korean Academy of Nursing. 2021;51(1).
37. Ping L. Problems and Countermeasures of medical insurance for urban and rural residents. Rural economy and technology*.* 2011; 29 (08): 216.
38. Announcing government information disclosure. 2021 May 7. In: Jiaxing Medical Insurance Bureau report [Internet]. Statistical express on the development of basic medical security in Jiaxing in 2020. [about 1 screen]. Available from: <http://ybj.jiaxing.gov.cn/art/2021/5/7/art_1229113711_58919368.html>
39. Zhu Chen. China's basic medical insurance coverage rate is nearly 95%, covering 1.27 billion people. Decision making reference of hospital leaders. 2011; 17(03).
40. Schoen C, Doty M. Inequities in access to medical care in five countries: findings from the 2001 common wealth fund international health policy survey. Health Policy. 2004; 67: 309-322．
41. Kun Zou, Hong-Ying Li, Die Zhou, Zai-Jun Liao. The effects of diagnosis-related groups payment on hospital healthcare in China: a systematic review. BMC Health Services Research. 2020; 20(5).
42. Medical Security Bureau of Zhejiang Provincial. Interim Measures for DRGs points payment of hospitalization expenses of basic medical insurance in Zhejiang Province . Bulletin of Zhejiang Provincial People's government. 2019; 28: 30-35.
43. Smith CA, Wright D, Day S. Distancing the mad: Jarvis’s law and the spatial distribution of admissions to the hamilton lunatic asylum in Canada, 1876 -1902. Social Science and Medicine. 2007; 1: 2362-2377.
44. Buor D. Distance as a predominant factor in the utilization of health services in the Kumasi Metropolis, Ghana. Geojournal. 2002; 56.
45. Teke K, Kisa A, Demir C. Appropriateness of admission and length of stay in a Turkish military hospital. Journal of Medical Systems. 2004; 28.
