# Supplementary material for: Relationship between outpatients’ sociodemographic and belief characteristics and their healthcare-seeking behavioral decision-making: Evidence from Jiaxing city, China
Source: PLoS One. 2022 Jun 30;17(6):e0270340. doi: 10.1371/journal.pone.0270340 (PMC9246228; doi:10.1371/journal.pone.0270340)
Supplement: Attachment — Submitted filename: Response_to_Reviewers.docx [file pone.0270340.s006.docx]

1) the additional information describing the machine learning techniques is satisfactory, however, I think the first revision includes too much detail in the text of the article, So, you went from too little in the original submission to too much in the revision in response to Reviewer 1. I suggest be edited so it can be a stand-alone amplification of the material in the main text and be incorporated with your response to Reviewer 1's comment 4 (4.Forth, what is artificial intelligence?...) and include that in a supplemental file. I've highlighted the details that I suggest could go into a supplemental file in the attached version of your revised manuscript.

Response:

Thanks for your suggestions. In this revised main text, we presented the machine learning part separately in the introduction of the decision tree analysis and included a more detailed explanation in this supplemental file.

In the main text on page 11:

Machine learning is concerned with features and labels. Features are observable attributes of the observed object, and labels are what we want to predict. In this research, the main decision tree task was to extract useful features and construct the mapping from features to labels; in other words, to draw a portrait.

2) I think Reviewer 1's comments 1 (First, there were misunderstanding of socio-demographic... ), 2 (Second, the author's understanding of medical insurance...)  and 5 (Last, the severity of the disease was determined by who...), and Revewer 2's comment 2 (One of my greatest concerns with this study...) raise important issues that should be available to readers who are interested, but are too detailed for the main text. Please edit your responses to those comments so they can be stand-along amplifications of the main text and include them, with the references, in the supplemental file that I suggested above. Please add references to this supplemental information at appropriate places in the main text.

Response:

For the Reviewer 1's comments 1, 2, 5 and Reviewer 2’s comment 2, in the revised main text, we have added some information, and more details are put in the points 1 to 4 of supplemental file. Moreover, we added references to the supplemental file by endnote in the main text.

3) Figure 1 that the software package produces won't be acceptable for publication. Please prepare another version of Figure 1 following PLOS ONE guidelines for figures. I think an acceptable figure could be prepared in Microsoft powerpoint and then converted to tif format using the PACE graphics processor (see PLOS ONE figure guidelines).

Response:

According to the requirements of PLOS ONE, we have modified the format of Figure 1.

3) The data supplement requires a variable key that explains the variable names, the data values with references to the corresponding questions in the Questionnaire. In other words, it requires a brief codebook. I think this could be added as an additional tab in the spreadsheet.

Response:

According to the requirements, we have added additional tabs in the spreadsheet of Table 1.

4) Parts of the revised manuscript don't conform to standard English usage, so I suggest the manuscript be reviewed by someone who is fluent in academic English prose.

Response:

According to the requirements, we invited Editage (www.editage.com) for the English language editing.

6) I have a number of other questions and suggestions regarding the revised manuscript.  I have attached a copy of the revised manuscript with my comments, questions and suggestions. Please address the following issues:

a) The explanation for overcrowding in tertiary hospitals and policy responses in the Introduction (pp 4-5) seems to say that the 2015 THDS was adopted to address a problem created by the  2009 Health Policy Reform. Please clarify.

Response:

Thank you for your comments that we have considered and incorporated the changes in the revised main text. Before 2009 in China, the 15% mark-up policy permitted by the government on drug prices allowed public hospitals to retain 15% profits of drug prices after selling to patients. Hence, as public hospitals, they could be self-sufficient at a large cost, and financial subsidies account for a small part of their income. The 2009 Health Policy Reform included a zero-mark-up drug policy and reduced prices for diagnostic tests. The purpose was to solve the problem that has plagued China for a long time: it is expensive to visit a doctor. The 2015 THDS is trying to solve the problem of overcrowding in tertiary hospitals.

In the revised manuscript, we have added some explanations regarding the policy on page 4.

b) In the Theoretical Model section (pp 6 and 7) the theoretical model that underlies the statistical analysis seems to be conflated with the statistical model and estimation technique. Provide more justification and explanation why what seem to be three different things can be described as one 'hybrid model', or distinguish them as different components of the analysis.

Response:

Thank you for your rigorous scientific commands. After careful consideration, we conflated the technology of machine learning and the theoretical model. Hence, in the revised main text, we deleted the machine learning from the 'hybrid theoretical framework'. The 'theoretical framework' included Andersen’s behavioral model of health services use and prospect theory. Please see this change in the main text on the page 6-7 in the revised manuscript. We also included the machine learning part separately in the introduction of decision tree analysis and a more detailed explanation in the supplemental file. Please see this change on the page 11 in the revised manuscript.

b) I think the relationship between the Patient Characteristics and Questionnaire Design sections is confusing and needs some reorganization or additional language to clarify

Response:

c) Thank you for your valuable comments. We have moved the section describing the demographic features to the Survey and Questionnaire Design section. Please see it on the page 8-9 in the revised manuscript.

d) Table 1 and its discussion should be moved the the Results section.

Response:

Thank you for your suggestions, we have moved the Table 1 and its discussion to the

Results section. Please see it on the page 14-15 in the revised manuscript.

e) Reference 27 at the bottom of page 12 (Taeho Jo, Machine Learning Foundations) describing the C4.5 algorithm isn't useful for readers not expert in machine learning (I can't find the term 'C4.5' in the text. The text does clearly use C4.5 or a closely related algorithm, but the general reader won't know this). I have suggested an alternative reference for C4.5, (and also the SVM and MLP algorithms and their relationships to decision trees), or clarify that the reference describes how that type of algorithm works, but don't suggest that the reference mentions C4.5 by name)

Response:

Thanks for your suggestions, and we have replaced the reference as you suggested: Moreno-Ibarra, Villuendas-Rey, Lytra et al. Classification of Diseases Using Machine Learning Algorithms: A Comparative Study. A Comparative Study. Mathematics 2021, 9, 1817. <https://doi.org/10.3390/math9151817.> We have added the introduction of C4.5 algorithm according to the reference: This is a decision tree type classification algorithm, and this type of classifier is among the most commonly used in classifying patterns, please see it on the page 11 in the revised manuscript; And added ‘the two classifiers were compared by the sensitivity or specificity to the predictive performance in machine learning’, please see it on the page 12 in the revised manuscript.

d) I think the description on pp 13-14 of  the logistic regression analysis says that it is used to validate the decision tree estimates, but also to compare and contrast (as in a sensitivity or robustness analysis?) the results of the two methods. I think this  is confusing and frankly can't be right. I think you are using logistic regression for two distinct functions that it cannot perform simultaneously. I would like clarification of how the logistic regression analysis is used to strengthen the decision tree analysis. I think its use should be characterized as a sensitivity or robustness analysis. Please see my comments in the attachment for more details.

Response:

Thank you for your valuable comments. As per your recommendation, we have changed this statement in our main text: Decision tree and logistic regression are two different classification methods. In many studies, with the same datasets, the two classifiers were compared by the sensitivity or specificity to the predictive performance in machine learning. Lee Yoonju got the common major predictors of suicide attempts in the two methods, showing the robustness of results using the two methods. Hence, we did the logistic regression analysis to compare and advance the results of the decision tree, with the datasets of questionnaires. Please see it on the page 12 in the revised manuscript.

e) The Analysis of the data from the Population Health Platform on pp 14-15 needs clarification. A clearer explanation of how it relates to the decision tree analysis is needed at the beginning of the section. I think how the Health Platform data is used to confirm the decision tree and logistic regression analysis becomes clear by the end of the presentation of the results later in the paper, but this should be made clear in the Materials and Methods section.

Response:

Thank you for your valuable comments. As per your recommendation, we have reinforced the explanation of the relationship between the two analyses and the necessity of Health Platform data analysis: The previous two analyses used the questionnaire survey data were based on the decision-making of patients' behavior. The survey was limited by the possible inconsistency of patients' external expression and behavioral consciousness and its scope in the city was representative but not comprehensive. Moreover, if different data and methods get similar results, it shows that they are robust. Hence, to verify our conclusions from the questionaries were reliable, we further analyzed additional data available through Jiaxing’s population health platform of outpatients who had visited the city’s hospitals. Please see it on the page 13 in the revised manuscript.
