# Supplementary material for: Relationship between outpatients’ sociodemographic and belief characteristics and their healthcare-seeking behavioral decision-making: Evidence from Jiaxing city, China
Source: PLoS One. 2022 Jun 30;17(6):e0270340. doi: 10.1371/journal.pone.0270340 (PMC9246228; doi:10.1371/journal.pone.0270340)
Supplement: Attachment — Submitted filename: Response_to_Reviewers.docx [file pone.0270340.s007.docx]

 1) Page 11: Statistical package implementation used to estimate the decision tree. PyCharm is referenced as the 'modeling procedure' (though I think the 'implementation of the decision tree analysis' is better). but PyCharm is a Python programming interface, not an estimation algorithm.  I think the most common implementation of the decision tree algorithm in Python is scikit-learn in the scikit package. However, I know of several custom implementations available on the internet that can be downloaded. I think the official term for things like sci-kit in Python is 'plug-in'. So, the way the tree must have been estimated was to instruct Python to run a specific decision tree estimator plug-in through the PyCharm interface. Please reference the specific plug-in  or source of the code, used to estimate the tree. If I am wrong, please give a reference that shows how you can estimate a tree tree using PyCharm by itself.

Response:

Thank you for your rigorous scientific commands. After careful consideration, we added the description of Sklearn, a Python’s machine learning package, and emphasized that the Pycharm is a tool rather than modeling procedure. Please see this change on the page 11 in the revised manuscript.

2) Page 21: In the Discussion section at the top of the page, you say "Economic factors were the most important for outpatients’ healthcare-seeking behavioral decision-making in China, despite having or not having medical insurance  or belonging to a specific profession." This statement seems to contradict the decision tree analysis where the first decision branch, or split, is whether the subject has health insurance or not. Could you please clarify? Is the discussion at the top of page 21 about more general population behavior in China for seeking any kind of health care? If so what is the source for that information? And how does that seemingly inconsistent piece of information relate the conclusion of the paper, why do you mention it?

Response:

As for "Economic factors were the most important for outpatients’ healthcare-seeking behavioral decision-making in China, despite having or not having medical insurance  or belonging to a specific profession", based on the previous analyses in the manuscript, we considered the following aspects: 1) From the text of "those without medical insurance and minor diseases were more likely to choose small hospitals" on page 19, we thought the choices maybe for economic factors (having not medical insurance means more money to see a doctor); 2) From the results of population health platform analysis about "outpatients without medical insurance and employed as farmers were more likely to choose primary and secondary than tertiary hospitals" and "This difference may be because of the farmer or non-farmer profession, as the proportion of farmers with medical insurance is lower than that of other professions" on page 17, we also thought the connection between having/not having medical insurance and profession was ultimately an economic factor; 3) From the text of "Financial status is important in our research. However, to respect patients’ privacy, their profession was used as a proxy for income (e.g., farmer, industrial worker, staff or civil servant, freelancer or individual owner), given that, in China, they are correlated to some extent [25]" on page 9, we also saw the connection of profession and economic factors.

So we can conclude that in developing country like China, outpatients’ healthcare-seeking behaviors are still firstly constrained by economic factors despite having or not having medical insurance  or belonging to a specific profession.

Style and production issues

1) Some of the references mix up first and last names of the authors. For example, reference 20 in the main text, the author Amos Tversky is listed as 'Amos T'. The author should be referred to as 'Tversky A'.  Please make sure all the references list the author name property.

Response:

Thank you for your correction. According to the requirements of PLOS ONE, we have changed some references, please see it on page 24-29.

2) Please do another check for typographical errors in the text. For example, at the top of page 11 'maching learning' appears instead of 'machine learning'.

Response:

Thank you for your careful check. We completed typographical errors checking for the whole paper.

3) Please review the text for consistent terminology, especially in the Discussion. For example, at the top of page 20, you say  "In contrast, farmers and industrial workers are more inclined to avoid seeking the highest standard of care." The contrast is with other types of people who seek care at large hospitals. Does 'highest standard of care' refer to 'large hospitals"?  And does 'large hospitals' refer to 'tertiary hospitals"? If so, exactly the same term should be used, otherwise please clarify the contrast. One of the main points of the paper is that large, tertiary hospitals are not really the 'highest standard of care' for minor conditions because other types of hospitals can provide equivalent standard of care for many health problems. I think a review of manuscript is needed to ensure that a clear standard terminology is used to refer to the most important terms used. This will prevent confusion, especially for readers who are not familiar with the Chinese health care system.

Response:

Thank you for your correction. In China, residents generally do not pay attention to the official classification of hospitals. Generally speaking, the large hospitals in their minds are often tertiary hospitals, and the small hospitals are primary and secondary hospitals. In the revised manuscript, we added these sentences on page 9. In addition, according to your suggestion, since our paper focused on building the Tiered-network Healthcare Delivery System, and the purpose of which was the diversion of the three level hospitals, we changed the large hospitals into tertiary hospitals and the small hospitals into primary and secondary hospitals, except for those parts related to the questionnaire survey.

4)All the programming languages and specific routines need to be referenced. The references can be very simple. For Stata, the reference I use is  'StataCorp (2017). Stata version 15. College Station, Texas.'

Response:

Thank you for your suggestion. According to the requirements, we have added the references of the programming languages, please see it on the page 27-28.

5) The figure for the decision tree is at the end of the main text. For production the style guide says to indicate where in the text you want the figure placed, with the title and description of the Figure. So you should place the contents of the supplemental file 'The annotation of Figure 1' where you want the figure to appear.

Response:

Thank you for your suggestion. We did that according to your requirement.

6) Add a variable key under Table 2 that explains the meaning of the variables, for example, of 'Prof-3'

Response:

Thank you for your suggestion. We added two variable keys under Table 2.

7) Thank you for preparing the supplemental file. I think the way you reference the different sections of the supplemental file in the main text is nonstandard and will confuse readers. I did check the style guidelines and the journal is not clear about what system is used to reference supplemental material. I recommend that you change how you reference the supplemental file in the main text to avoid last minute problems in production. For example, at the bottom of page 7, where you have a superscript 'i' I recommend that you simply replace that with the text '(See section S1 of the Supplemental File for more details) ', in normal font (not superscript). Note that the journal requires that  the number of any section, or table or figure in supplemental files must be preceded by an S).

Response:

Thank you for your suggestion. We did that according to your requirement.

8) in the the Figure of the decision tree, I don't see where the definitions of the three target classifications (A, B, C) are defined. I believe that 'C' refers to tertiary hospitals but I'm not sure. I recommend that A, B, and C be defined in the main text, and clearly related to the terms you use for each health facility, and include that information in the Annotation for Figure 1. Also, the journal if vague on numbering of Figures. When there is only one figure, I can't find in the style guide whether it should be referred to as "Figure' or 'Figure 1'. In any case, if you decide to refer l as 'Figure 1' then make sure that all references to that figure is consistent, including the name of the  file you upload.

Response:

I am sorry for confusing you about that. A and C in this manuscript just meant the difference of options, and we gave them specific meanings on page 9. In the Annotation for Figure 1, we added the information about A and C, please see it on page 16.
